# P-1591. The Rate of Bacterial/Fungal Co-infections and Secondary Infections in Hospitalized COVID-19-infected patients at a Tertiary Care Hospital in Oman

**DOI:** 10.1093/ofid/ofaf695.1770

**Published:** 2026-01-11

**Authors:** Fatiya Al Salml

**Affiliations:** OMSB, Al-seeb, Masqat, Oman

## Abstract

**Background:**

This study aimed to estimate the rate of co-infections and secondary infections among adult patients infected with COVID-19. Furthermore, to describe the clinical characteristics and the risk factors for patients with/without co-infections and secondary infections and to understand the pathogens associated with these infections.

**Figure:**
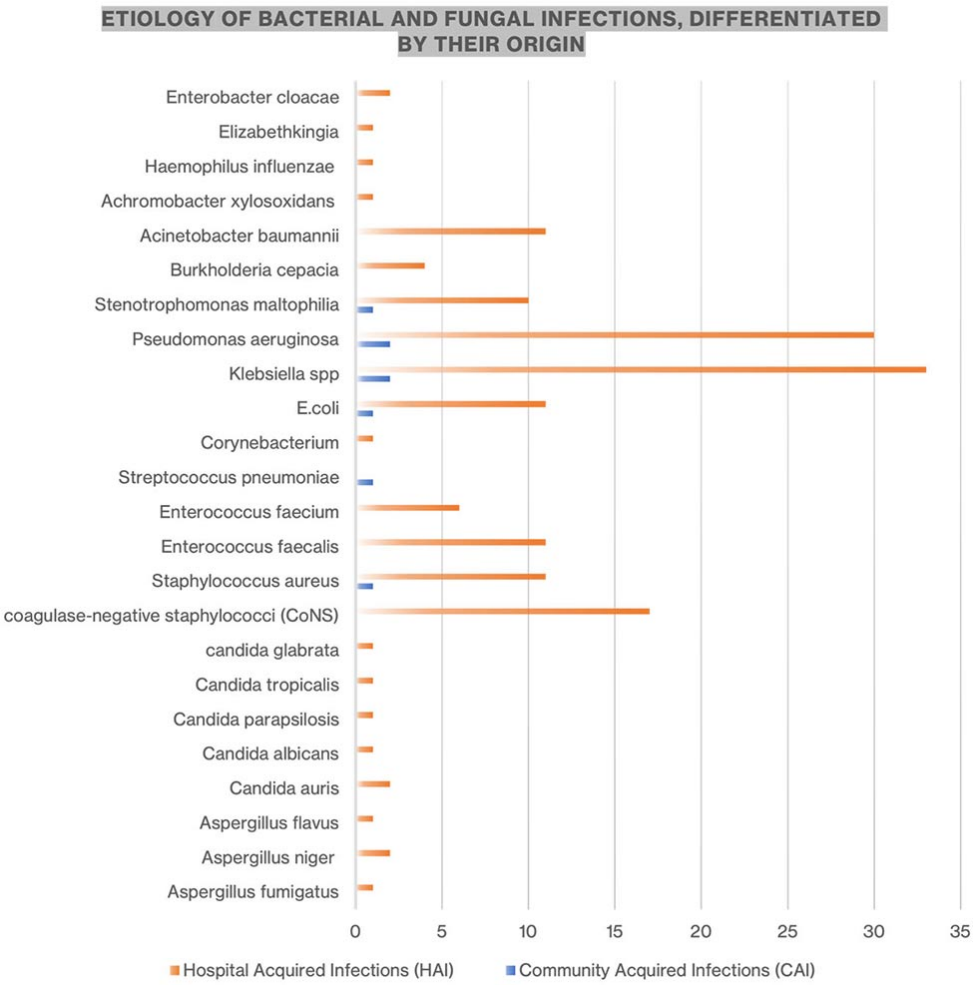

**Figure:**
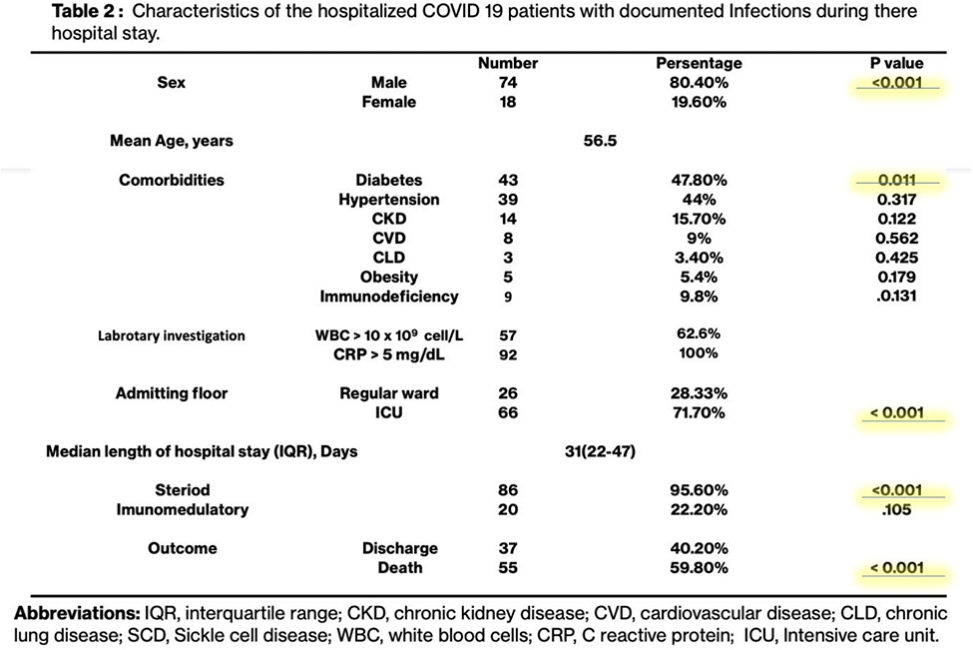

**Methods:**

This study was a single-center, retrospective observational study. We included all laboratory-confirmed COVID-19-infected adult patients admitted at Sultan Qaboos University Hospital from March 2020 to December 2022. Demographic, clinical, and microbiological

data were collected retrospectively from the hospital information system (Track-care and lab-track)

.

**Results:**

A total of 373 adult patients with COVID-19 infection were included. One hundred thirteen episodes of infections were found in 92 patients with a total prevalence of 24.7 (95% CI: 20.37‒29.36). Community-acquired infections were 2.1%, whereas hospital-acquired infections accounted for 22.8%. The majority of patients had at least one comorbidity, visited the intensive care unit, and received

steroids as part of treatment for COVID-19 pneumonia. Overall, 92% were bacterial infections and 8% fungal. *Pseudomonas aeruginosa* and *Klebsiella spp* were the most common organisms isolated for healthcare-associated infections. The fungal infections identified were hospital-acquired, and candidemia was the most common infection caused by different species. The overall mortality rate was 60% in hospitalized patients with superinfections.

**Conclusion:**

To better understand the rate of co-infections, diagnostic algorithms are needed for early detection and management. Further studies are suggested to understand the contributing factors to co-infections and superinfections in the Omani population.

**Disclosures:**

All Authors: No reported disclosures

